# Evaluation of a water extract of So-Cheong-Ryong-Tang for acute toxicity and genotoxicity using *in vitro* and *in vivo* tests

**DOI:** 10.1186/s12906-015-0737-x

**Published:** 2015-07-16

**Authors:** Mee-Young Lee, Chang-Sebo Seo, Ji-Young Kim, Hyeun-Kyoo Shin

**Affiliations:** Herbal Medicine Formulation Research Group, Korea Institute of Oriental Medicine, 483 Expo-ro, Yusung-gu, Daejeon, 305-811 South Korea; Division of Nonclinical Studies, Korea Institute of Toxicology, P.O. Box 123, 19 Sinseongro, Yuseong-gu, Daejeon, 305-343 South Korea

**Keywords:** So-Cheong-Ryong-Tang, Ames test, Chromosome aberration assay, Micronucleus assay, Acute toxicity

## Abstract

**Background:**

So-Cheong-Ryong-Tang, a traditional Korean medicine, has been used empirically for the treatment of asthma, allergic rhinitis, and colds for hundreds of years. However, its genotoxicity has been rarely examined.

**Methods:**

We therefore investigated the genotoxicity of an aqueous extract of So-Cheong-Ryong-Tang (SCRT) in two *in vitro* and one *in vivo* assays: a bacterial reverse mutation assay (Ames test), a chromosomal aberration assay, and a micronucleus assay, respectively.

**Results:**

In the bacterial reverse mutation assay, SCRT did not increase revertant colony numbers in *Salmonella. typhimurium* strains (TA98, TA100, TA1535, and TA1537) or an *Escherichia coli* strain (WP2 *uvrA*) regardless of metabolic activation or the duration of treatment. However, statistically significant differences in the incidence of chromosomal aberrations following SCRT >4000 μg/mL were observed in Chinese hamster lung cells exposed with or without an S9 enzyme and cofactor mixture.

**Conclusions:**

These results suggest further genotoxic testing of SCRT, such as a comet assay, to ascertain its generally recognized safety.

**Electronic supplementary material:**

The online version of this article (doi:10.1186/s12906-015-0737-x) contains supplementary material, which is available to authorized users.

## Background

The traditional herbal medicine So-Cheong-Ryong-Tang (known as Xiao-Qing-Long-Tang in traditional Chinese medicine and as Sho-seiryu-to in Japanese Kampo medicine) is a mixture of eight herbal preparations (*Herba Ephedrae*, *Radix Paeoniae*, *Fructus Schisandrae*, *Tuber Pinelliae*, *Asiasari Radix*, *Rhizoma Crudus Zingiberis*, *Ramulus Cinnamomi*, and *Radix et Rhizoma Glycyrrhizae*). This mixture has long been used to treat allergic rhinitis, bronchitis, and bronchial asthma [1]. So-Cheong-Ryong-Tang has several pharmacological effects such as antiallergic activity on airway inflammation in a mouse model [2] and decreasing antigen-induced numbers of eosinophils and basophils [3].

The use of herbal prescriptions as primary therapeutics or supplements for improving health-related conditions is popular worldwide [4]. Traditional herbal medicines are selected to accentuate the therapeutic activity of their components, while attenuating the toxicity or side effects of components from other herbs in the mixture [5]. During the past two decades, studies have demonstrated the potential efficacy of SCRT as a treatment for asthma in mouse [1, 2] and guinea pig [6] models, and in clinical studies. To determine the safety of medicines, systematic toxicological studies must be performed using experimental models to predict toxicity and to set criteria for selecting a safe dose in humans. Despite the popular use of So-Cheong-Ryong-Tang in traditional Korea medicine, no systematic evaluation of its genotoxic effects has been performed. Assessment of the genotoxic properties of folk medicine is important because damage to genetic material may lead to critical mutations and may thereby increase the risk of diseases including cancer [7]. Therefore, the purpose of the present study was to evaluate the safety of an aqueous extract of So-Cheong-Ryong-Tang (SCRT) and its genotoxicity and acute toxicity. We assessed these properties using the standard battery of tests recommended by the Korea Food and Drug Administration (KFDA): the bacterial reverse mutation test (Ames test), the chromosome aberration test, and the *in vivo* micronucleus test.

## Methods

### Reagents

Coumarin and cinnamic acid were purchased from Sigma-Aldrich (St Louis, MO, USA). Albiflorin, paeoniflorin, cinnamaldehyde, glycyrrhizin, and schizandrin were obtained from Wako Pure Chemical Industries Ltd (Osaka, Japan). Liquiritin was purchased from NPC BioTechnology (Daejeon, Korea). The purity of each compound was determined as ≥98 % by HPLC analysis. HPLC-grade reagents, water, methanol, and acetonitrile were obtained from J.T. Baker (Phillipsburg, NJ, USA). Acetic acid was purchased from Junsei (Tokyo, Japan). The materials for preparing the aqueous extract of SCRT were purchased from Kwangmyungdang Medicinal Herbs (Ulsan, Korea). Voucher specimens (2012-KE13-1 to KE13-8) have been deposited at the Herbal Formulation Research Group, Korea Institute of Oriental Medicine (KIOM).

### Preparation of standard and sample solutions

**Fig. 1:**
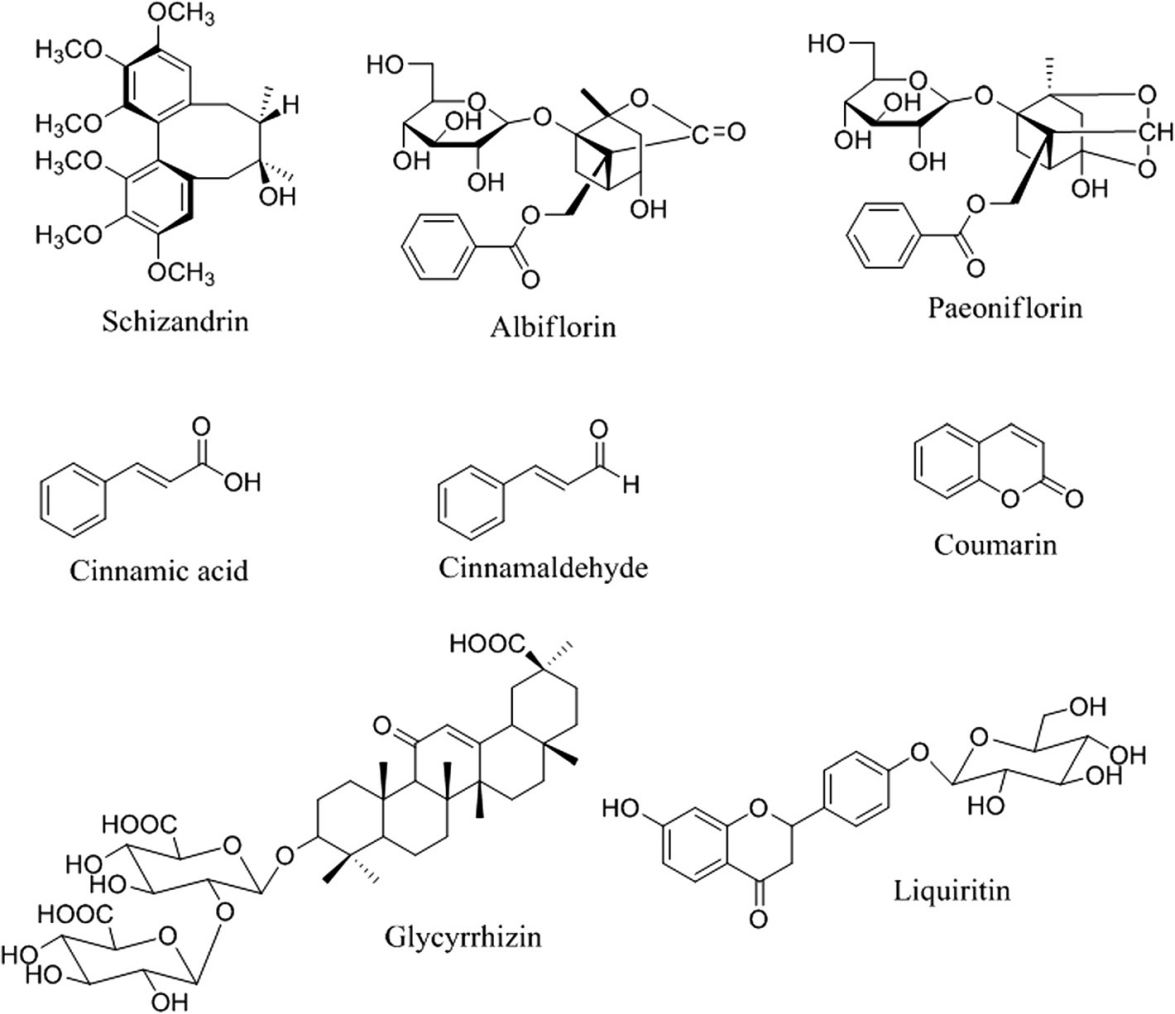
Chemical structures of the eight main compounds found in SCRT

Standard stock solutions of eight compounds, albiflorin, paeoniflorin, liquiritin, coumarin, cinnamic acid, cinnamaldehyde, glycyrrhizin, and schizandrin (Figure 1) were dissolved in methanol at concentrations of 1 mg/mL and stored below 4 °C.

SCRT was formulated from eight herbs (Additional file 1: Table S1, 72.0 kg) and was extracted with distilled water at 100 °C for 2 h in herb extractor (COSMOS-660, Kyungseo Machine Co, Inchon, Korea). The extract was filtrated using a standard sieve (No. 270, 53 μm) and freeze-dried (9.3 kg). The yield of extract was 12.9 %. Lyophilized SCRT extract (200 mg) was dissolved in distilled water (20 mL). The solution was filtered through a SmartPor GHP syringe filter (0.2 μm pore size, Woongki Science, Seoul, Korea).

### HPLC conditions

We performed a simultaneous analysis of extract components using a Shimadzu LC-20A HPLC system (Shimadzu, Kyoto, Japan), consisting of a solvent delivery unit, an online degasser, a column oven, an autosampler, and a photodiode array (PDA) detector. A data processor employed LCsolution software (version 1.24). The analytical column used for separation was a Gemini C18 column (250 mm × 4.6 mm, particle size 5 μm; Phenomenex, Torrance, CA, USA) and was maintained at 40 °C. The mobile phases consisted of 1.0 % (v/v) aqueous acetic acid (A) and 1.0 % (v/v) acetic acid in acetonitrile (B). The gradient flow was as follows: (A)/(B) = 85/15 (0 min) → (A)/(B) = 35/65 (35 min) → (A)/(B) = 0/100 (45 min; hold for 5 min) → (A)/(B) = 85/15 (55 min; hold for 15 min). The analysis was conducted at a flow rate of 1.0 mL/min with PDA detection at 230 nm, 254 nm, and 280 nm. The injection volume was 10 μL.

### Acute oral toxicity test

To test the acute oral toxicity of SCRT, specific pathogen-free Sprague Dawley rats of both sexes were obtained at 5 weeks of age from Orient Bio Co. (Seongnam, Korea) and used after 1 week of quarantine and acclimatization. This study was approved by KIOM’s Institutional Animal Care and Use Committee (IACUC); it was performed at the Korea Institute of Toxicology (KIT; Daejeon, Korea) and conducted according to the guidelines of KIT’s IACUC, which is accredited by AAALAC International (1998) under the GLP Regulations for Nonclinical Laboratory Studies. A preliminary study showed that a single oral administration of SCRT did not induce any toxic effect at dose levels of 0 and 2000 mg/kg/day. Based on these results, a dose of 2000 mg/kg/day was selected as the toxicological limited dose recommended by the Organization for Economic Cooperation and Development (OECD) guideline (1997: Principles of Good Laboratory Practice). Healthy male and female rats were assigned to groups of five rats of each sex. SCRT was suspended in distilled water, and the volume for application of a dose of 10 mL/kg body weight was calculated. The vehicle control rats received an equivalent volume of distilled water only. All animals were observed, and mortality, clinical signs, body weight changes, and gross findings were recorded for 14 days.

### Bacterial reverse mutation assay (Ames test)

The experimental methods used in the study were based on the published reports by Maron and Ames [8, 9], with minor modifications. *Salmonella typhimurium* strains TA98 and TA1537 (to detect frame-shift mutagens), TA1535, and TA100, and *Escherichia coli* strain WP2 *uvrA* (to detect base pair-substitution mutagens) were obtained from Molecular Toxicology Inc. (Boone, NC, USA) and used as the test strains. The bacterial reverse mutation assay was performed as described previously [10].

### Chromosome aberration test

Chinese hamster lung (CHL) cells were obtained from the American Type Culture Collection (Manassas, VA, USA) in 2004. The chromosome aberration assay was performed according to OECD guideline No. 473 “*In Vitro* Mammalian Chromosome Aberration Test” [11] and previously performed methods with minor modification as described by Ishidate et al. [12] and Dean and Danford [13].

### *In vivo* micronucleus test

The preliminary study showed that oral administration of SCRT at a dose of 2000 mg/kg did not induce any toxic effect (data not shown). The highest dose was determined based on the dose range-finding study, and 2000 mg/kg, which was the limit dose for treatment up to 14 days according to the OECD guidelines, was selected as the maximum dose. Specific pathogen-free male CrljOri:CD1 (ICR) mice weighing 27.2–30.0 g were obtained from Orient Bio Co. (Seongnam, Korea) at 6 weeks old. Mice were used in experiments after 1 week of quarantine and acclimatization. This study was reviewed and assessed by KIT’s IACUC. This micronucleus test was conducted in accordance with OECD guideline No. 474 “Mammalian Erythrocyte Micronucleus Test” [14], with minor modifications. The micronucleus test using mice was performed as described previously [10].

### Statistical analyses

Body weights are presented as mean ± standard deviation (SD). All statistical analyses were performed with the Path/Tox System (version 4.2.2) using an *F* test. The statistical analyses for the *in vitro* chromosomal aberration results were conducted using a method described by Richardson et al. [15]. The number of aberrant metaphases (excluding gaps) and the number of [PP + ER] were analyzed. The Fisher’s exact test was performed to compare the vehicle control and to test item-treated groups. Fisher’s exact test was used to compare the vehicle and positive control groups. Differences were regarded as significant at *P* < 0.05. No statistical analysis was performed on the Ames test results. Statistical evaluation of the *in vivo* micronucleus results was performed using the method described by Lovell et al. [16] with minor modification. *In vivo* micronucleus statistical analysis was conducted as described previously [10].

## Results

### HPLC analysis

A chromatogram of SCRT was obtained using an HPLC-PDA. Under optimized chromatography conditions, eight constituents were eluted within 35 min in the control sample analysis using mobile phases comprising solvent A (1.0 %, v/v acetic acid in water) and solvent B (1.0 %, v/v acetic acid in acetonitrile).

**Fig. 2:**
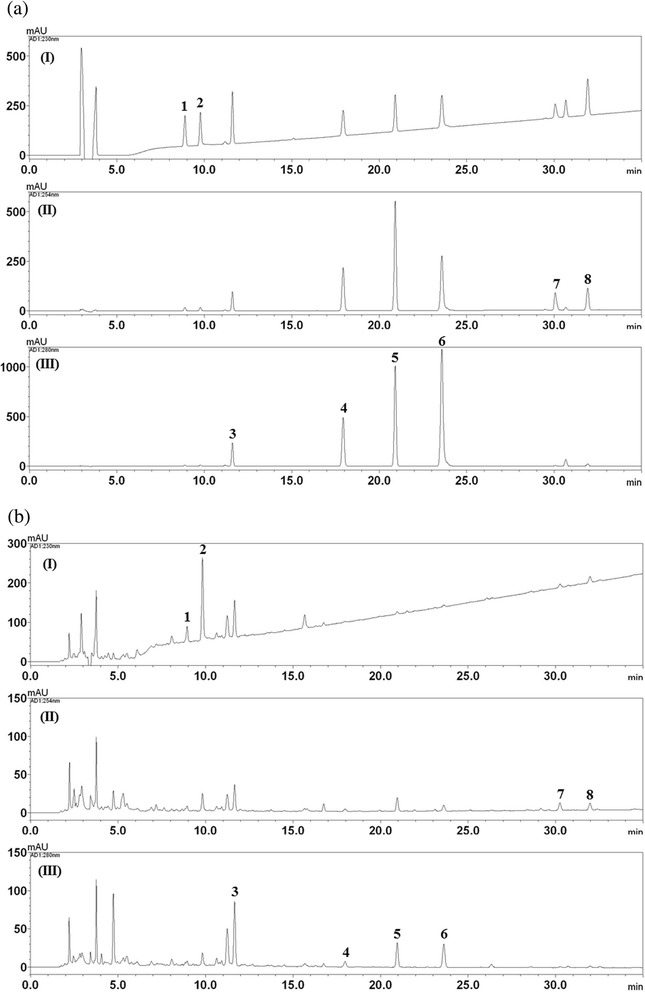
HPLC chromatograms of a standard mixture (**a**) and SCRT (**b**) at 230 nm (I), 254 nm (II), and 280 nm (III). Albiflorin (1), paeoniflorin (2), liquiritin (3), coumarin (4), cinnamic acid (5), cinnamaldehyde (6), glycyrrhizin (7), and schizandrin (8)

The linearity of the peak area (*y*) versus concentration (*x*, μg/mL) curve for each component was used to masure the contents of the main components in SCRT (Fig. 2a and b). Additional file 1: Table S2 showed the calibration curves and correlation coefficients (*r*^2^) of 8 constituents. The retention times of the 8 components, albiflorin, paeoniflorin, liquiritin, coumarin, cinnamic acid, cinnamaldehyde, glycyrrhizin, and schzandrin were 8.88 min, 9.76 min, 11.59 min, 17.83 min, 20.83 min, 23.45 min, 29.37 min, and 31.83 min, respectively. Fig. 2a and b shows the HPLC chromatogram of standard solution and water extract of SCRT. The contents of 8 compounds were 0.22–12.02 mg/g (Additional file 1: Table S3).

### Acute oral toxicity test

**Fig. 3:**
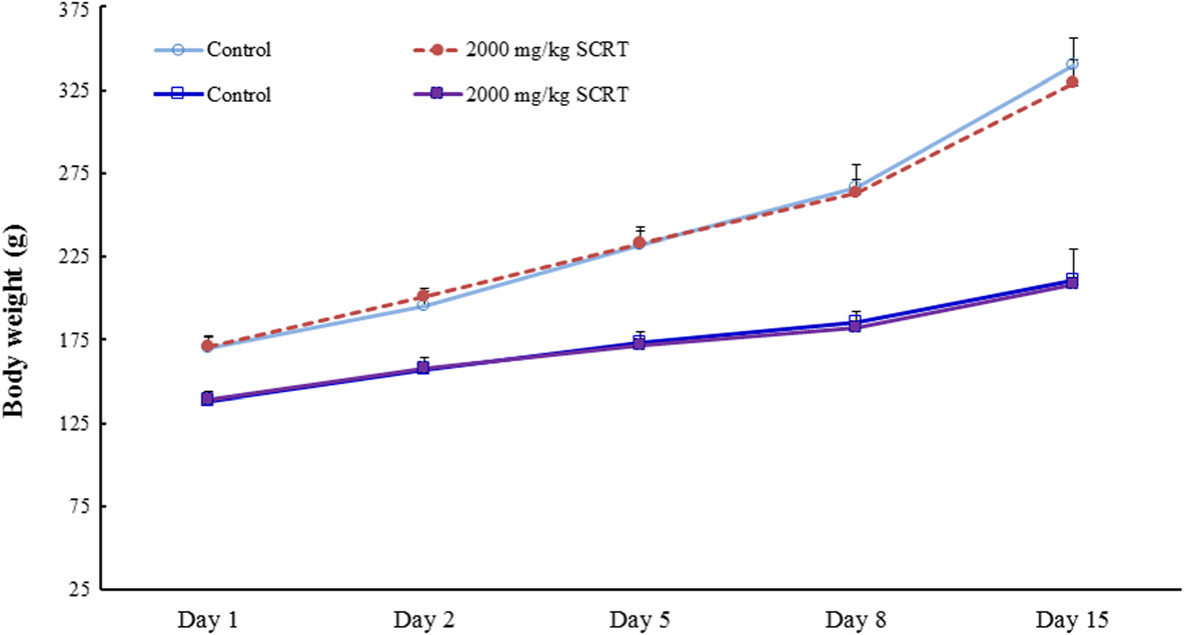
Changes of mean body weight in male and female rats after single oral administration of SCRT at dose levels of 0 (○) and 2000 mg/kg (●) in male rats and 0 (□) and 2000 mg/kg (■) in female rats. There were no significant differences in body weight between rats in the SCRT-treated and control groups. Values are presented as mean ± SD

No mortality or clinical symptoms of toxicity were observed in rats of either sex in any group during the 14-day observation period of the SCRT-treated group. The body weight changes are summarized in Fig. 3. For both sexes, the changes in body weight did not differ significantly between treated with 2000 mg/kg/day of SCRT and the vehicle control group. At the time of the scheduled autopsy, there were no abnormal observations including for the lung, heart, thymus, stomach, liver, adrenals, and spleen in the male or female rats given 2000 mg/kg/day of SCRT.

### Bacterial reverse mutation assay (Ames test)

**Fig. 4:**
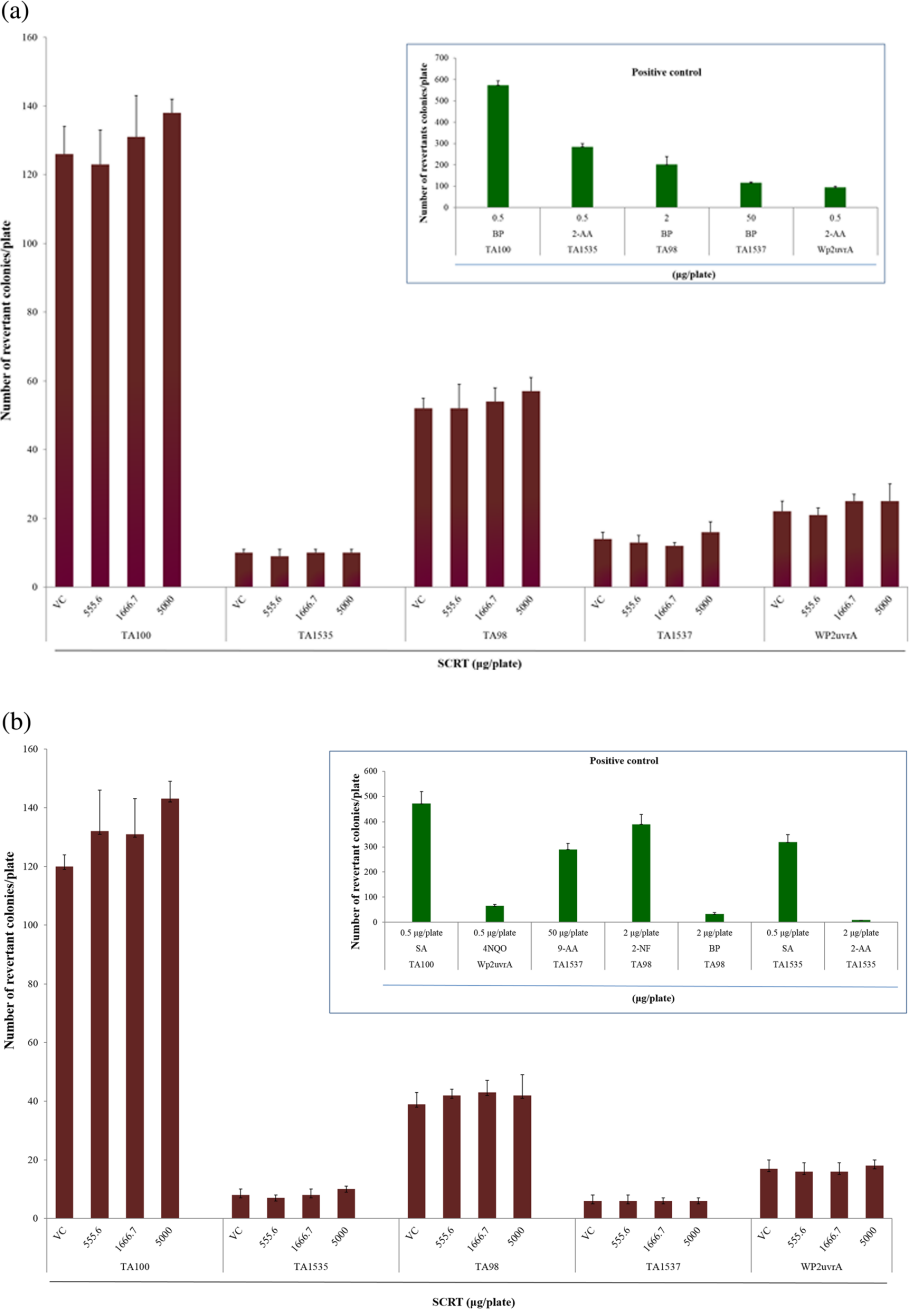
Effect of SCRT on bacterial reverse mutation assay (Ames test) (**a**) with (+S9 mix) and (**b**) without (−S9 mix) metabolic activation. BP: Benzo(a)pyrene, SA: Sodium azide, 2-AA: 2-Aminoanthracene, 2-NF: 2-Nitrofluorene, 4NQO: 4-Nitroquinoline *N-oxide*, 9-AA: 9-Aminoacridine, VC: vehicle control

The positive controls showed significantly increased numbers of revertant colonies, indicating that the assay was valid. No positive mutagenic response was observed in any of the *S. typhimurium* or *E. coli* strains tested compared with the concurrent vehicle control groups regardless of the presence (Fig. 4a) or absence (Fig. 4b) of the S9 mixture up to 5000 μg/plate.

### Chromosome aberration tests

According to our preliminary study (data not shown), SCRT neither inhibited cell growth nor killed CHL cells. We determined the concentration range (2000, 3000, 4000, 4500, and 5000 μg/mL) that was most compatible with a good cell-proliferating ability and that produced a sufficient number of metaphases for the confirmatory assay. Therefore, we used 5000 μg/mL as the highest exposure level and serial dilutions for further dose–response tests.

There was a statistically significant increase in the number of metaphase cells with structural aberrations at 6 h or 22 h with or without the S9 mixture in the SCRT-treated groups at 4000 μg/mL or 5000 μg/mL (Additional file 1: Table S4) compared with the vehicle control group (*P* < 0.01). In the positive control groups, there were significant increases in the number of aberrant metaphases. The number of metaphases with structural aberrations in the vehicle and positive control groups was within the range established in the historical data of KIT (KIT, 2009). These findings confirm that the methodologies used in this study were valid. Therefore, under the conditions of this test, SCRT showed a positive response in the chromosomal aberration test.

### Micronucleus test

No abnormal changes were observed in the general appearance or body weight between the first and final administrations in the vehicle control group, positive control group, or the groups treated with 500, 1000, or 2000 mg/kg/day of SCRT (Additional file 1: Table S5). The number of MNPCEs/2000 PCEs and PCE/(PCE + NCE) did not increase significantly in the groups treated with SCRT at 500, 1000, or 2000 mg/kg/day (Additional file 1: Table S6). There was a significant increase in the number of MNPCEs/2000 PCEs in the positive control group, indicating that the present study was performed under acceptable experimental conditions.

## Discussion and conclusions

The long history of herbal prescriptions seems that they are nontoxic and clinically effective. However, for present medicinal therapeutics, there have been few scientific studies undertaken to determine the safety of traditional medicinal herbs. Therefore, concerns have been raised about the lack of scientific evidence for the safety of herbal medicines [17]. SCRT was proven to be safe in a 90-day oral toxicity study in rats [18]. In a series of safety evaluations for SCRT, a genotoxicity test was conducted in the present work to check its capacity for mutagenicity. Here, we performed to detect chromosome aberrations in CHL cells, a bacterial reverse mutation test using the *S. typhimurium*/*E. coli* incorporation assay (Ames test), and an *in vivo* micronucleus test recommended by the KFDA. We evaluated acute toxicity using the standard battery of tests recommended by the KFDA. The present study was performed according to OECD guidelines for the testing of chemicals in accordance with modern Good Laboratory Practice Regulations.

In the acute toxicity test, a single oral dose of SCRT did not cause any adverse effects at doses of up to 2000 mg/kg/day. Genotoxicity tests have been used mainly for the prediction of carcinogenicity of compounds because compounds that are have the potential to cause carcinogenic and/or mutagenic effects in humans [19, 20].

The Ames test has been extensively used to evaluate mutagenic and carcinogenic risks. In the present Ames test, there was no positive mutagenic response at any concentration of SCRT up to 5000 μg/plate in any of the *S. typhimurium* (TA100, TA1535, TA98, and TA1537) or *E. coli* (WP2 *uvrA*) strains compared with the concurrent vehicle control groups regardless of the presence or absence of the metabolic activation system (S9 mixture). This indicates that SCRT is not mutagenic to bacterial strains TA98, TA100, TA1535, TA1537, and WP2 *uvrA*.

The *in vitro* chromosomal aberration test is a widely used assessment of genotoxicity. Many birth defects and human genetic diseases are associated with abnormal chromosome complements. In the chromosomal aberration test, statistically increases in the number of metaphases with structural aberrations at over 4000 μg/mL of SCRT were seen in the presence or absence of the metabolic activation system in CHL cells. The results of the *in vitro* chromosomal aberration assay suggest that SCRT can cause genetic disorders. However, SCRT consists of eight different herbs, and each herb contains various components, such as flavonoids. Some flavonoids can induce cellular mutagenecity [21]. Therefore, it is possible that the genotoxicity of SCRT observed in the chromosomal aberration test is related to a specific single component. An *in vivo* chromosomal aberration test is thus warranted.

The micronucleus test is used to detect mutagenic substances, thus altering the equitable distribution of chromosomes [22]. In the micronucleus test using ICR mice, no abnormal clinical signs in general appearance and body weight were observed in the 500, 1000, or 2000 mg/kg/day SCRT treatment groups. SCRT did not induce any significant increases in MNPCEs, and there was no significant decrease in the PCE/(PCE + NCE) ratio up to 2000 mg/kg in the SCRT treatment groups compared with the vehicle control. From these results, we conclude that SCRT did not induce mutagenesis under the conditions of this study.

In conclusion, SCRT had no genotoxic effects (in the Ames test and the *in vivo* micronucleus test) except in the chromosomal aberration test, suggesting that SCRT may cause mutations in chromosomes *in vitro*. Further detailed experiments are needed to identify whether SCRT contains any genotoxic component and, if so, the underlying mechanism(s) of genotoxicity.

## Additional file

Additional file 1: Table S1. The combination of crude components of SCRT. **Table S2.** Calibration curves of eight marker components (*n* = 3). **Table S3.** Contents of eight components in the SCRT by HPLC (*n* = 3). **Table S4.** Chromosome aberration assay and relative cell counts of SCRT. **Table S5.** Body weight changes of Micronucleus test in mice following administration of SCRT. Table S6. Micronucleus test in mice following a single oral dose of SCR.
