# Supplementary material for: Evaluation of a water extract of So-Cheong-Ryong-Tang for acute toxicity and genotoxicity using in vitro and in vivo tests
Source: BMC Complement Altern Med. 2015 Jul 16;15:235. doi: 10.1186/s12906-015-0737-x (PMC4502466; doi:10.1186/s12906-015-0737-x)
Supplement: Additional file 1: Table S1. — The combination of crude components of SCRT. Table S2. Calibration curves of eight marker components (n = 3). Table S3. Contents of eight components in the SCRT by HPLC (n = 3). Table S4. Chromosome aberration assay and relative cell counts of SCRT. Table S5. Body weight changes of Micronucleus test in mice following administration of SCRT. Table S6. Micronucleus test in mice following a single oral dose of SCR. [file 12906_2015_737_MOESM1_ESM.pptx]

## Slide 1
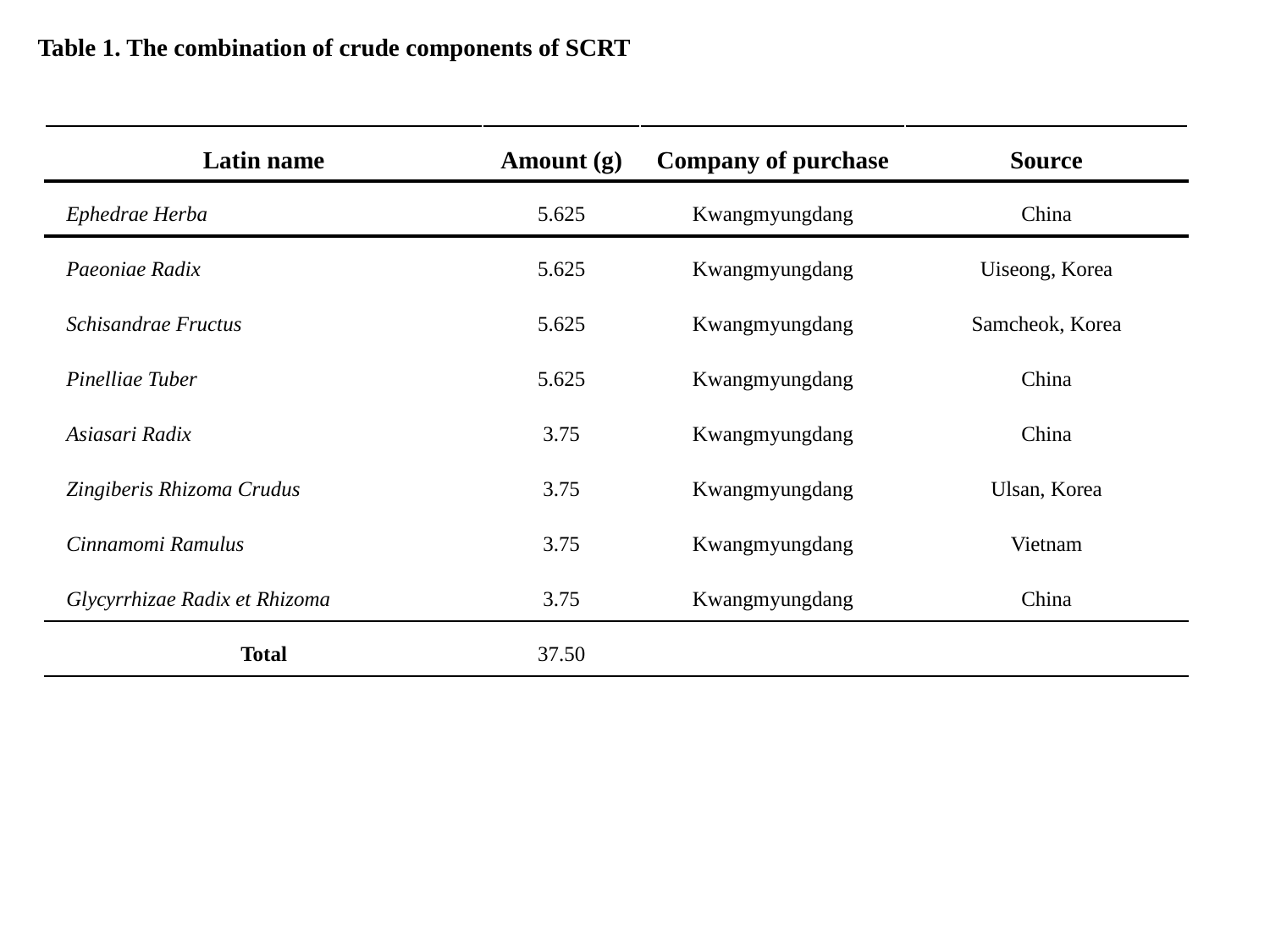

Table 1. The combination of crude components of SCRT
| Latin name | Amount (g) | Company of purchase | Source |
| --- | --- | --- | --- |
| Ephedrae Herba | 5.625 | Kwangmyungdang | China |
| Paeoniae Radix | 5.625 | Kwangmyungdang | Uiseong, Korea |
| Schisandrae Fructus | 5.625 | Kwangmyungdang | Samcheok, Korea |
| Pinelliae Tuber | 5.625 | Kwangmyungdang | China |
| Asiasari Radix | 3.75 | Kwangmyungdang | China |
| Zingiberis Rhizoma Crudus | 3.75 | Kwangmyungdang | Ulsan, Korea |
| Cinnamomi Ramulus | 3.75 | Kwangmyungdang | Vietnam |
| Glycyrrhizae Radix et Rhizoma | 3.75 | Kwangmyungdang | China |
| Total | 37.50 | | |

## Slide 2
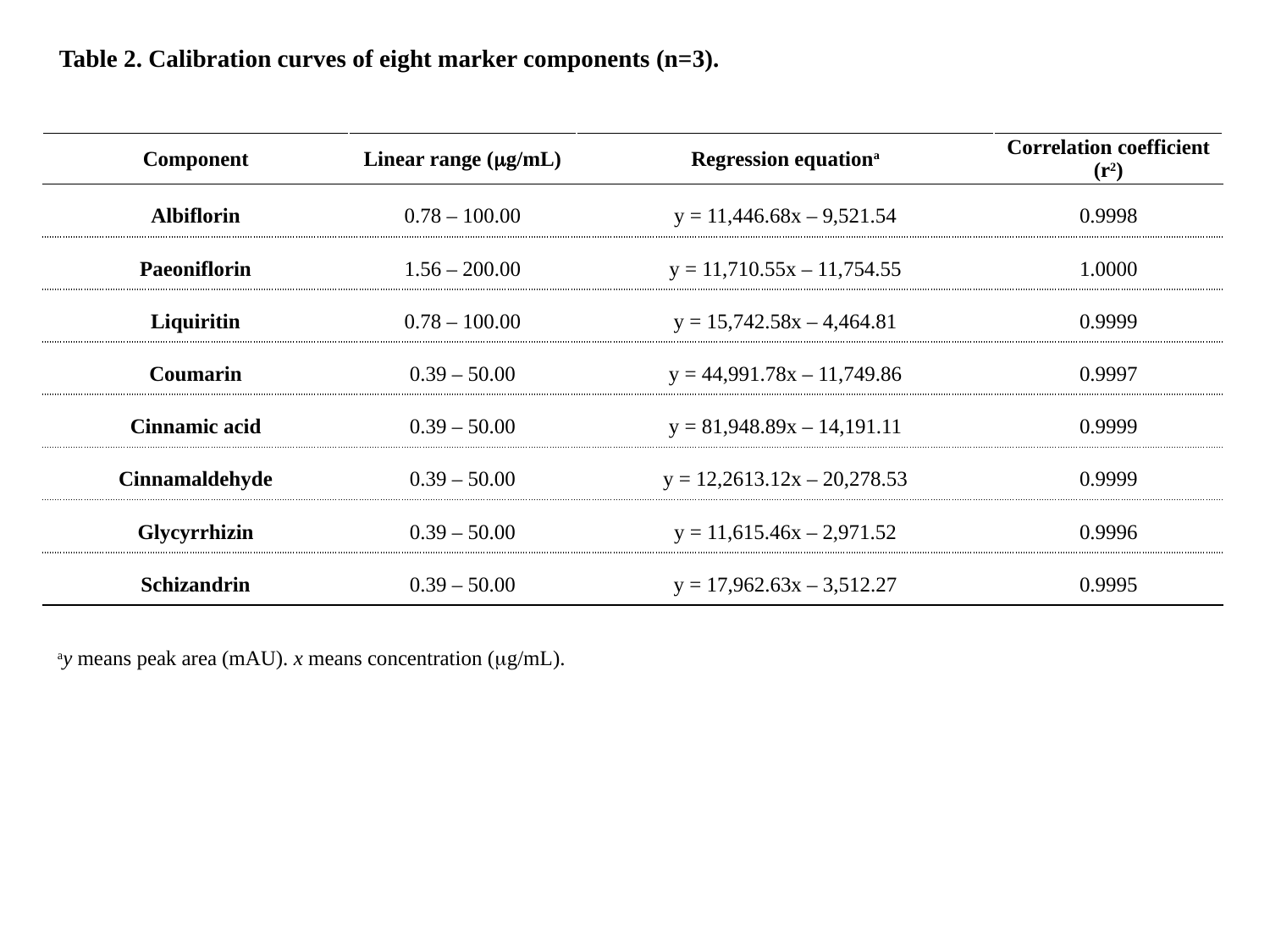

Table 2. Calibration curves of eight marker components (n=3).
| Component | Linear range (μg/mL) | Regression equationa | Correlation coefficient (r2) |
| --- | --- | --- | --- |
| Albiflorin | 0.78 – 100.00 | y = 11,446.68x – 9,521.54 | 0.9998 |
| Paeoniflorin | 1.56 – 200.00 | y = 11,710.55x – 11,754.55 | 1.0000 |
| Liquiritin | 0.78 – 100.00 | y = 15,742.58x – 4,464.81 | 0.9999 |
| Coumarin | 0.39 – 50.00 | y = 44,991.78x – 11,749.86 | 0.9997 |
| Cinnamic acid | 0.39 – 50.00 | y = 81,948.89x – 14,191.11 | 0.9999 |
| Cinnamaldehyde | 0.39 – 50.00 | y = 12,2613.12x – 20,278.53 | 0.9999 |
| Glycyrrhizin | 0.39 – 50.00 | y = 11,615.46x – 2,971.52 | 0.9996 |
| Schizandrin | 0.39 – 50.00 | y = 17,962.63x – 3,512.27 | 0.9995 |
ay means peak area (mAU). x means concentration (μg/mL).

## Slide 3
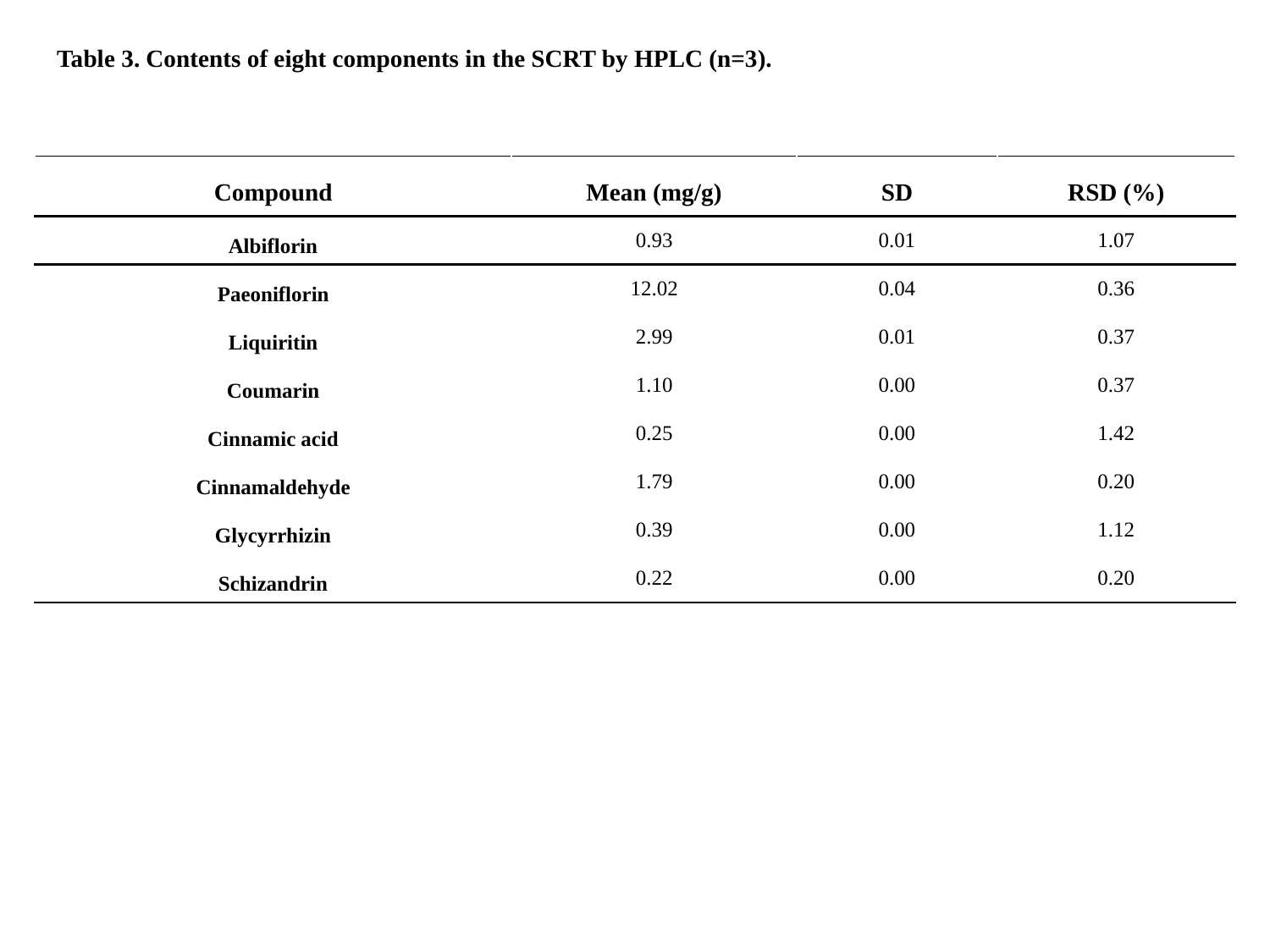

Table 3. Contents of eight components in the SCRT by HPLC (n=3).
| Compound | Mean (mg/g) | SD | RSD (%) |
| --- | --- | --- | --- |
| Albiflorin | 0.93 | 0.01 | 1.07 |
| Paeoniflorin | 12.02 | 0.04 | 0.36 |
| Liquiritin | 2.99 | 0.01 | 0.37 |
| Coumarin | 1.10 | 0.00 | 0.37 |
| Cinnamic acid | 0.25 | 0.00 | 1.42 |
| Cinnamaldehyde | 1.79 | 0.00 | 0.20 |
| Glycyrrhizin | 0.39 | 0.00 | 1.12 |
| Schizandrin | 0.22 | 0.00 | 0.20 |

## Slide 4
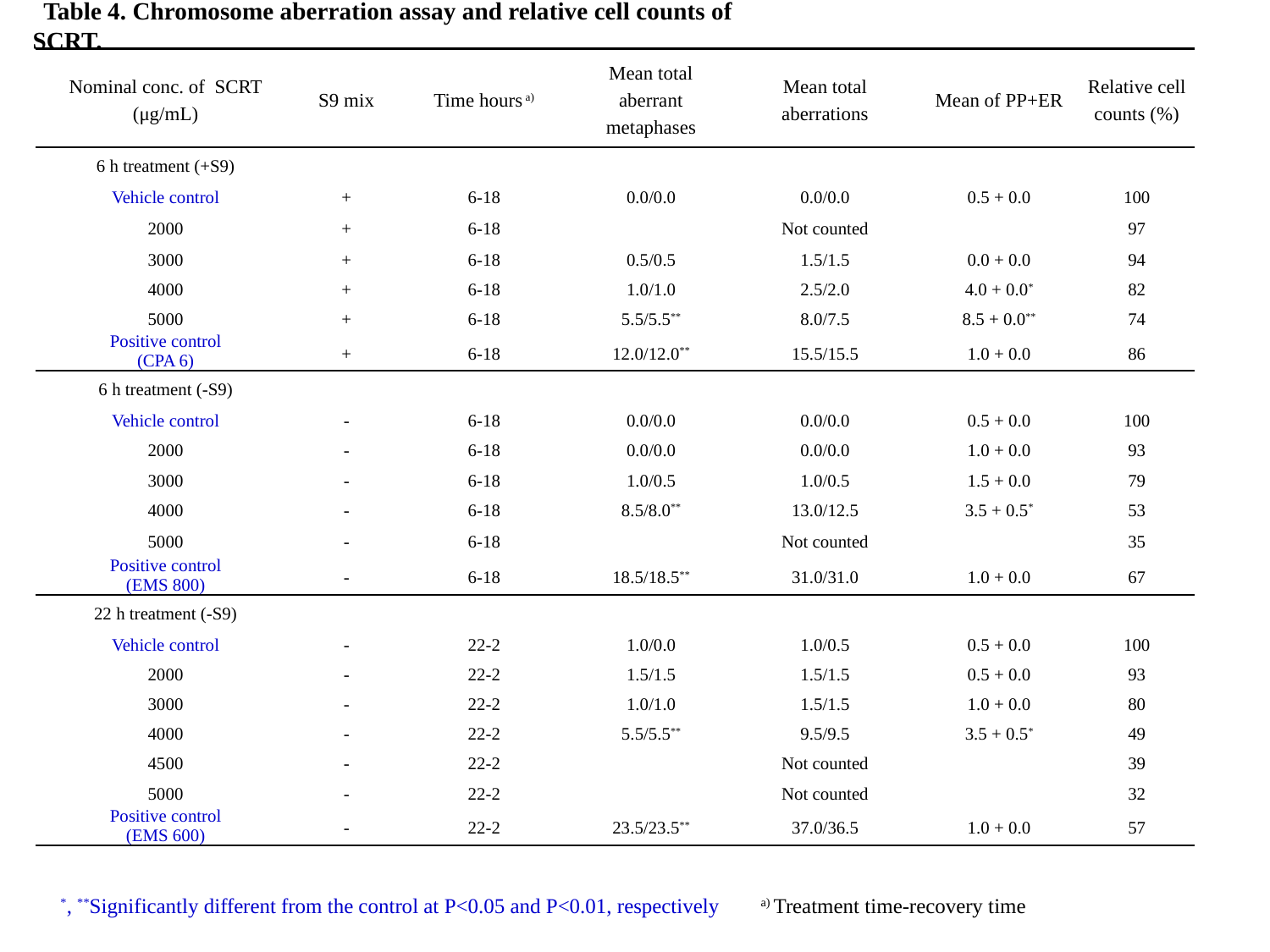

Table 4. Chromosome aberration assay and relative cell counts of SCRT.
| Nominal conc. of SCRT (μg/mL) | S9 mix | Time hours a) | Mean total aberrant metaphases | Mean total aberrations | Mean of PP+ER | Relative cell counts (%) |
| --- | --- | --- | --- | --- | --- | --- |
| 6 h treatment (+S9) | | | | | | |
| Vehicle control | + | 6-18 | 0.0/0.0 | 0.0/0.0 | 0.5 + 0.0 | 100 |
| 2000 | + | 6-18 | | Not counted | | 97 |
| 3000 | + | 6-18 | 0.5/0.5 | 1.5/1.5 | 0.0 + 0.0 | 94 |
| 4000 | + | 6-18 | 1.0/1.0 | 2.5/2.0 | 4.0 + 0.0\* | 82 |
| 5000 | + | 6-18 | 5.5/5.5\*\* | 8.0/7.5 | 8.5 + 0.0\*\* | 74 |
| Positive control (CPA 6) | + | 6-18 | 12.0/12.0\*\* | 15.5/15.5 | 1.0 + 0.0 | 86 |
| 6 h treatment (-S9) | | | | | | |
| Vehicle control | - | 6-18 | 0.0/0.0 | 0.0/0.0 | 0.5 + 0.0 | 100 |
| 2000 | - | 6-18 | 0.0/0.0 | 0.0/0.0 | 1.0 + 0.0 | 93 |
| 3000 | - | 6-18 | 1.0/0.5 | 1.0/0.5 | 1.5 + 0.0 | 79 |
| 4000 | - | 6-18 | 8.5/8.0\*\* | 13.0/12.5 | 3.5 + 0.5\* | 53 |
| 5000 | - | 6-18 | | Not counted | | 35 |
| Positive control (EMS 800) | - | 6-18 | 18.5/18.5\*\* | 31.0/31.0 | 1.0 + 0.0 | 67 |
| 22 h treatment (-S9) | | | | | | |
| Vehicle control | - | 22-2 | 1.0/0.0 | 1.0/0.5 | 0.5 + 0.0 | 100 |
| 2000 | - | 22-2 | 1.5/1.5 | 1.5/1.5 | 0.5 + 0.0 | 93 |
| 3000 | - | 22-2 | 1.0/1.0 | 1.5/1.5 | 1.0 + 0.0 | 80 |
| 4000 | - | 22-2 | 5.5/5.5\*\* | 9.5/9.5 | 3.5 + 0.5\* | 49 |
| 4500 | - | 22-2 | | Not counted | | 39 |
| 5000 | - | 22-2 | | Not counted | | 32 |
| Positive control (EMS 600) | - | 22-2 | 23.5/23.5\*\* | 37.0/36.5 | 1.0 + 0.0 | 57 |
*, **Significantly different from the control at P<0.05 and P<0.01, respectively
 a) Treatment time-recovery time

## Slide 5
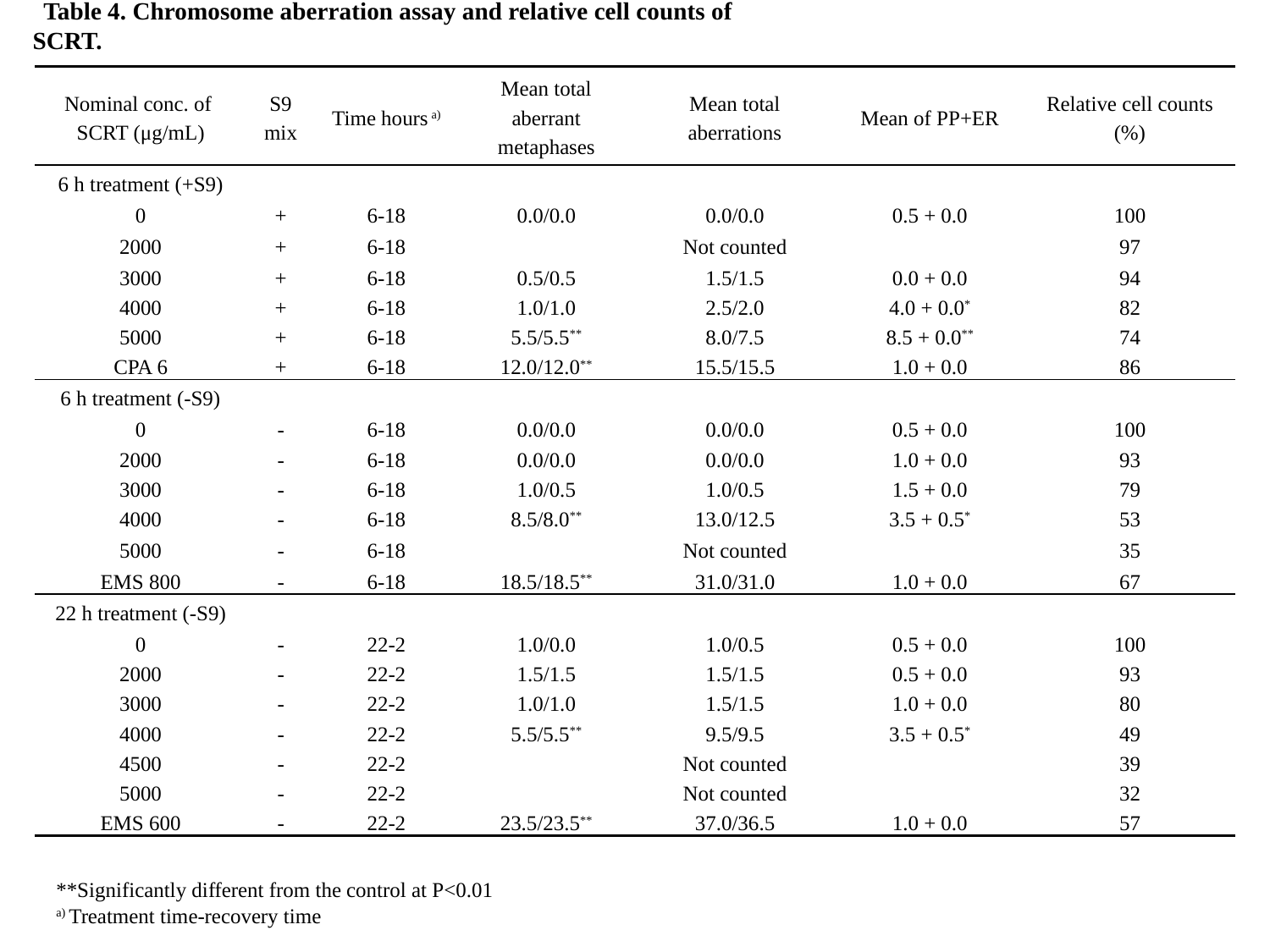

Table 4. Chromosome aberration assay and relative cell counts of SCRT.
| Nominal conc. of SCRT (μg/mL) | S9 mix | Time hours a) | Mean total aberrant metaphases | Mean total aberrations | Mean of PP+ER | Relative cell counts (%) |
| --- | --- | --- | --- | --- | --- | --- |
| 6 h treatment (+S9) | | | | | | |
| 0 | + | 6-18 | 0.0/0.0 | 0.0/0.0 | 0.5 + 0.0 | 100 |
| 2000 | + | 6-18 | | Not counted | | 97 |
| 3000 | + | 6-18 | 0.5/0.5 | 1.5/1.5 | 0.0 + 0.0 | 94 |
| 4000 | + | 6-18 | 1.0/1.0 | 2.5/2.0 | 4.0 + 0.0\* | 82 |
| 5000 | + | 6-18 | 5.5/5.5\*\* | 8.0/7.5 | 8.5 + 0.0\*\* | 74 |
| CPA 6 | + | 6-18 | 12.0/12.0\*\* | 15.5/15.5 | 1.0 + 0.0 | 86 |
| 6 h treatment (-S9) | | | | | | |
| 0 | - | 6-18 | 0.0/0.0 | 0.0/0.0 | 0.5 + 0.0 | 100 |
| 2000 | - | 6-18 | 0.0/0.0 | 0.0/0.0 | 1.0 + 0.0 | 93 |
| 3000 | - | 6-18 | 1.0/0.5 | 1.0/0.5 | 1.5 + 0.0 | 79 |
| 4000 | - | 6-18 | 8.5/8.0\*\* | 13.0/12.5 | 3.5 + 0.5\* | 53 |
| 5000 | - | 6-18 | | Not counted | | 35 |
| EMS 800 | - | 6-18 | 18.5/18.5\*\* | 31.0/31.0 | 1.0 + 0.0 | 67 |
| 22 h treatment (-S9) | | | | | | |
| 0 | - | 22-2 | 1.0/0.0 | 1.0/0.5 | 0.5 + 0.0 | 100 |
| 2000 | - | 22-2 | 1.5/1.5 | 1.5/1.5 | 0.5 + 0.0 | 93 |
| 3000 | - | 22-2 | 1.0/1.0 | 1.5/1.5 | 1.0 + 0.0 | 80 |
| 4000 | - | 22-2 | 5.5/5.5\*\* | 9.5/9.5 | 3.5 + 0.5\* | 49 |
| 4500 | - | 22-2 | | Not counted | | 39 |
| 5000 | - | 22-2 | | Not counted | | 32 |
| EMS 600 | - | 22-2 | 23.5/23.5\*\* | 37.0/36.5 | 1.0 + 0.0 | 57 |
**Significantly different from the control at P<0.01
 a) Treatment time-recovery time

## Slide 6
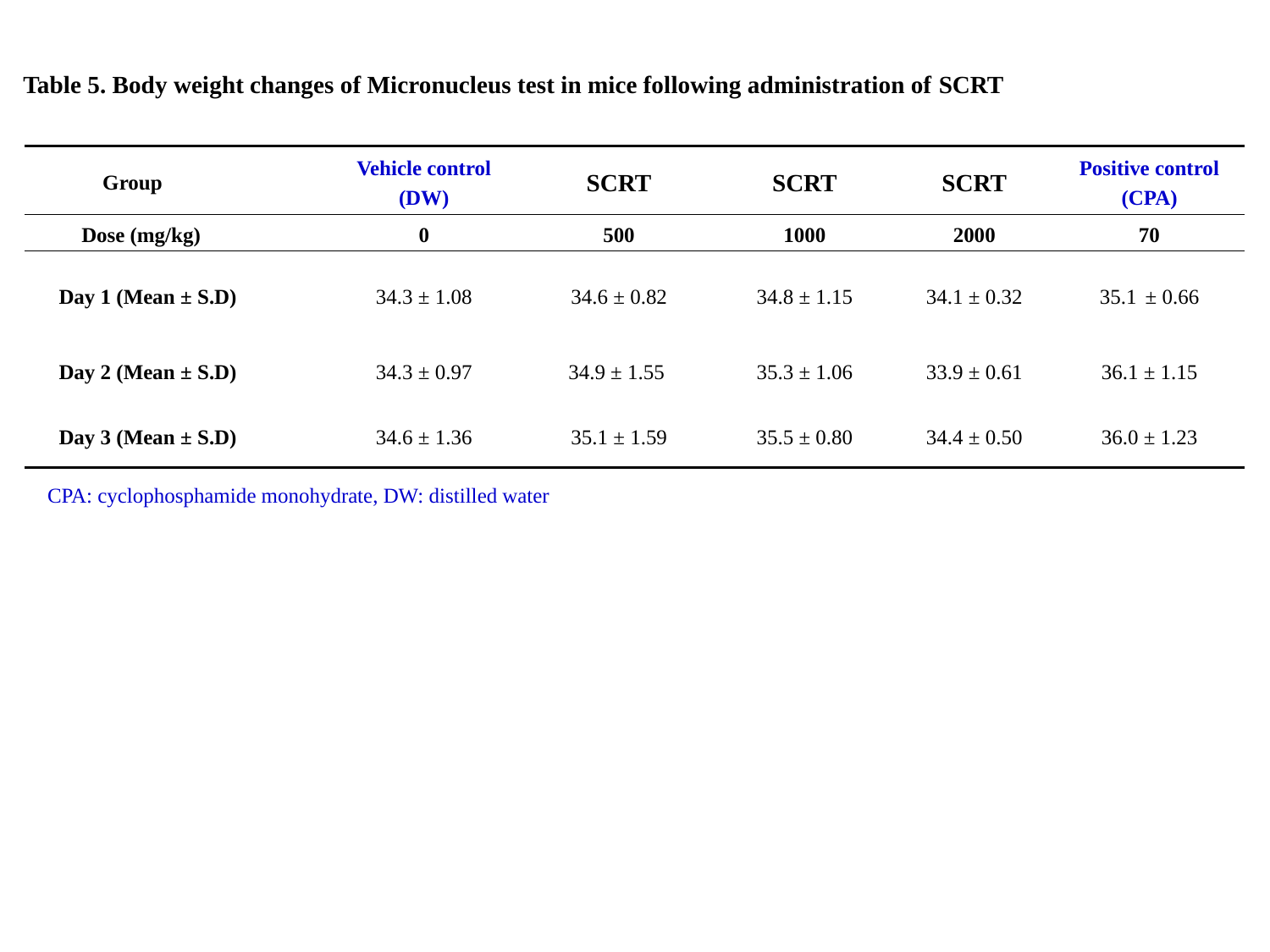

Table 5. Body weight changes of Micronucleus test in mice following administration of SCRT
| Group | Vehicle control (DW) | SCRT | SCRT | SCRT | Positive control (CPA) |
| --- | --- | --- | --- | --- | --- |
| Dose (mg/kg) | 0 | 500 | 1000 | 2000 | 70 |
| Day 1 (Mean ± S.D) | 34.3 ± 1.08 | 34.6 ± 0.82 | 34.8 ± 1.15 | 34.1 ± 0.32 | 35.1 ± 0.66 |
| Day 2 (Mean ± S.D) | 34.3 ± 0.97 | 34.9 ± 1.55 | 35.3 ± 1.06 | 33.9 ± 0.61 | 36.1 ± 1.15 |
| Day 3 (Mean ± S.D) | 34.6 ± 1.36 | 35.1 ± 1.59 | 35.5 ± 0.80 | 34.4 ± 0.50 | 36.0 ± 1.23 |
CPA: cyclophosphamide monohydrate, DW: distilled water

## Slide 7
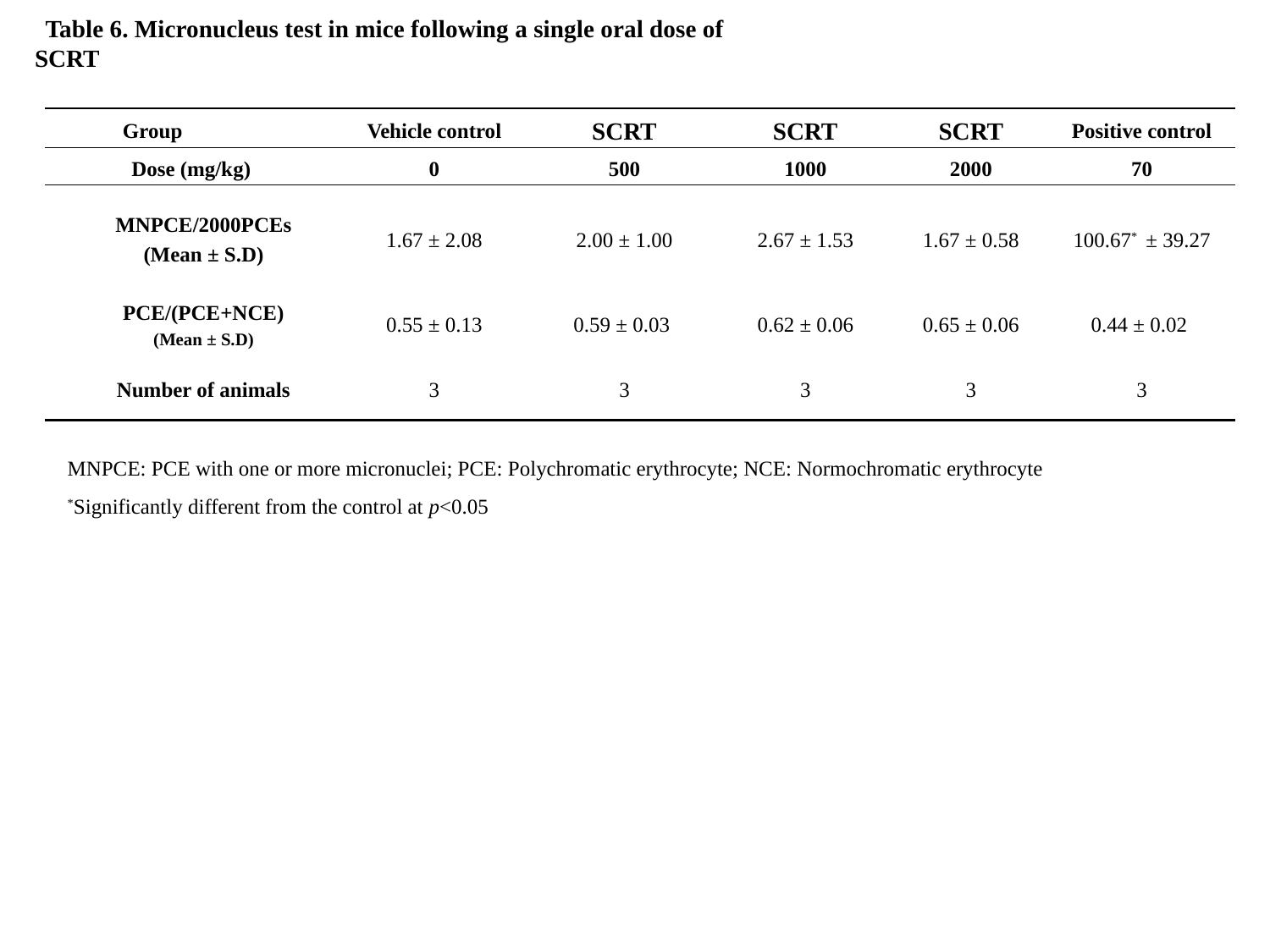

Table 6. Micronucleus test in mice following a single oral dose of SCRT
| Group | Vehicle control | SCRT | SCRT | SCRT | Positive control |
| --- | --- | --- | --- | --- | --- |
| Dose (mg/kg) | 0 | 500 | 1000 | 2000 | 70 |
| MNPCE/2000PCEs (Mean ± S.D) | 1.67 ± 2.08 | 2.00 ± 1.00 | 2.67 ± 1.53 | 1.67 ± 0.58 | 100.67\* ± 39.27 |
| PCE/(PCE+NCE) (Mean ± S.D) | 0.55 ± 0.13 | 0.59 ± 0.03 | 0.62 ± 0.06 | 0.65 ± 0.06 | 0.44 ± 0.02 |
| Number of animals | 3 | 3 | 3 | 3 | 3 |
MNPCE: PCE with one or more micronuclei; PCE: Polychromatic erythrocyte; NCE: Normochromatic erythrocyte
*Significantly different from the control at p<0.05
